# Genome-Wide Transcriptome Profiling of *Mycobacterium* *smegmatis* MC^2^ 155 Cultivated in Minimal Media Supplemented with Cholesterol, Androstenedione or Glycerol

**DOI:** 10.3390/ijms17050689

**Published:** 2016-05-07

**Authors:** Qun Li, Fanglan Ge, Yunya Tan, Guangxiang Zhang, Wei Li

**Affiliations:** College of Life Sciences, Sichuan Normal University, Chengdu 610101, China; liqun01234@163.com (Q.L.); gefanglan@tom.com (F.G.); 080603060b12@sina.com (Y.T.); zhgx-163@163.com (G.Z.)

**Keywords:** *Mycobacterium smegmatis*, RNA-Seq, cholesterol, mammalian cell entry

## Abstract

*Mycobacterium smegmatis* strain MC^2^ 155 is an attractive model organism for the study of *M. tuberculosis* and other mycobacterial pathogens, as it can grow well using cholesterol as a carbon resource. However, its global transcriptomic response remains largely unrevealed. In this study, *M. smegmatis* MC^2^ 155 cultivated in androstenedione, cholesterol and glycerol supplemented media were collected separately for a RNA-Sequencing study. The results showed that 6004, 6681 and 6348 genes were expressed in androstenedione, cholesterol and glycerol supplemented media, and 5891 genes were expressed in all three conditions, with 237 specially expressed in cholesterol added medium. A total of 1852 and 454 genes were significantly up-regulated by cholesterol compared with the other two supplements. Only occasional changes were observed in basic carbon and nitrogen metabolism, while almost all of the genes involved in cholesterol catabolism and mammalian cell entry (MCE) were up-regulated by cholesterol, but not by androstenedione. Eleven and 16 gene clusters were induced by cholesterol when compared with glycerol or androstenedione, respectively. This study provides a comprehensive analysis of the cholesterol responsive transcriptome of *M. smegmatis*. Our results indicated that cholesterol induced many more genes and increased the expression of the majority of genes involved in cholesterol degradation and MCE in *M.* *smegmatis*, while androstenedione did not have the same effect.

## 1. Introduction

Cholesterol is a terpenoid lipid formed by a carbon skeleton and four fused alicyclic rings. It plays an essential role as a structural component of animal cell membranes [1]. Cholesterol is frequently found in the biosphere with great relevance in biology, medicine and chemistry, not only because of its natural abundance, but also due to its high resistance to microbial degradation. Therefore, the catabolism of cholesterol by specific bacteria has attracted considerable attention, in part as a potential means of producing bioactive derivatives from this steroid [2,3].

As the leading cause of tuberculosis (TB), *Mycobacterium tuberculosis* can enter monolayers of HeLa, monkey kidney, human amnion cells and lung epithelial cells [4,5]. It uses different parts of the cholesterol molecule as the sole carbon source for energy and also for the biosynthesis of phthiocerol dimycocerosate (PDIM), a virulence-associated lipid [6]. Other mycobacteria and members of the *Nocardia*, *Rhodococcus*, and *Streptomyces* genera show the same ability to degrade cholesterol and have the potential to synthesize new steroid derivatives [7,8]. Biochemical and genetic studies have revealed that a suite of genes involved in cholesterol degradation are critical for the survival of *M. tuberculosis* in macrophages [9,10]. The *MCE4* operon encodes a specific cholesterol uptake system in cholesterol degrading bacteria [6,11,12,13]. The subsequent pathway responsible for the aerobic degradation of cholesterol can be divided into four major steps [3]. The first step involves the oxidation of cholesterol to cholest-5-en-3-one followed by the isomerization to cholestenone [14,15]. The degradation of the alkyl side-chain can be initiated by the cytochromes Cyp125 [16,17] or Cyp142 [18,19], followed by a β-oxidation-like process initiated by an ATP-dependent sterol/steroid CoA ligase [16,20,21]. The third step is central catabolism. Once the aliphatic chain has been oxidized, androstenedione (AD) is converted to androsta-1,4-diene-3,17-dione (ADD) [22,23,24], followed by a sequential transformation yielding 2-hydroxyhexa-2,4-dienoic and 9,17-dioxo-1,2,3,4,10,19-hexanorandrostan-5-oic (DOHNAA) acids [3,10]. The last step involves the degradation of 2-hydroxyhexa-2,4-dienoic DOHNAA acids leading the metabolites to enter central metabolic pathways, however, the corresponding process remains largely uncharacterized [3].

Unlike *M. tuberculosis*, *M. smegmatis* is a non-pathogenic member of the genus *Mycobacterium*. It is not dependent on animal cells for growth, but is frequently found in soil, water and plants. Although it shares more than 2000 gene homologs with *M. tuberculosis* and shares the same unusual cell wall structure of *M. tuberculosis* and other mycobacterial species [25], *M. smegmatis* grows much more rapidly than other species. *M. smegmatis* strain MC^2^ 155 is a mutant of *M. smegmatis* [26]. It can be much more efficiently transformed with plasmid vectors using electroporation (10 to 100 thousand times) than other strains of this species. Therefore, *M. smegmatis* MC^2^ 155 has been developed as a very attractive model organism for research on *M. tuberculosis* and other mycobacterial pathogens in the laboratory, including genetic analyses and investigations of cholesterol catabolism.

Uhía *et al.* found that 89 *M. smegmatis* genes were up-regulated at least three fold when cells were grown on cholesterol compared with that growing on glycerol by using microarray [27], and 39 of the catabolic genes were organized into three specific clusters. However, there is only one study done in *M. smegmatis* and microarrays show some limitations on throughput and noise ratios [28]. A more analysis is urgently needed to investigate the genome-wide cholesterol response in this model bacterium. The inception of RNA-sequencing technology (RNA-Seq) in 2005 provides a new method to uncover transcriptomic data with a much higher throughput than previous technology [29]. It shows major advantages in robustness, resolution and inter-lab portability over several microarray platforms [30]. In the current study, *M. smegmatis* strain MC^2^ 155 was cultured in chemically defined media supplemented with androstenedione, cholesterol or glycerol. Total RNAs were extracted from three corresponding samples and submitted to next-generation sequencing (NGS) platform for RNA-Seq study. The global expression patterns were carefully analyzed to characterize the genome-wide cholesterol response, and also whether androstenedione, a derivative of cholesterol, can stimulate the same response.

## 2. Results

### 2.1. RNA Sequencing and Global Expression Patterns

**Figure 1:**
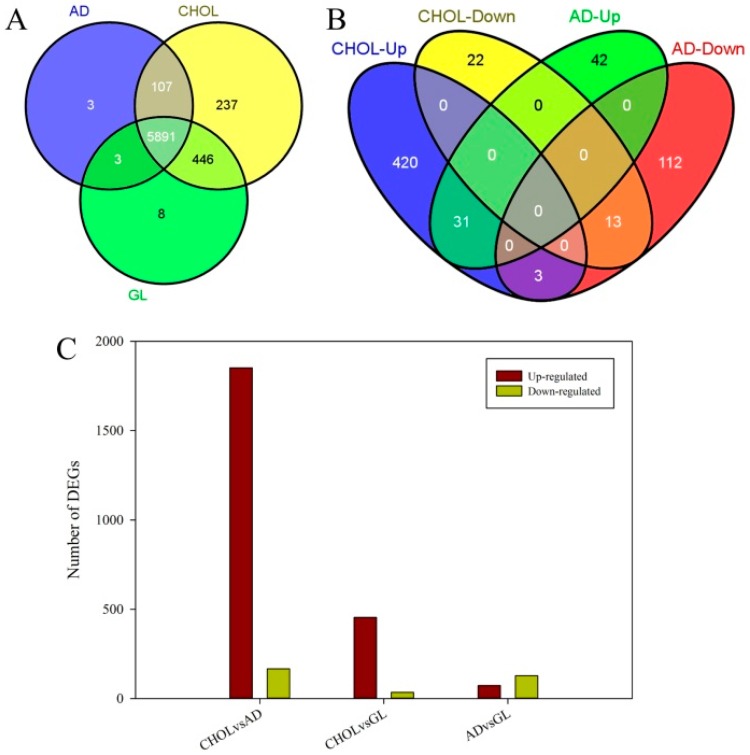
Genes expression profiling in different *M.* *smegmatis* samples. (**A**) Overlap examinations were performed basing on the resulting gene lists of three samples by VENNY [33]; (**B**) overlap examinations of differentially expressed genes (DEGs) identified between cholesterol and glycerol, androstenedione and glycerol; (**C**) DEGs identified pair-wisely among three samples. AD, CHOL and GL correspond to three different conditions: 20 mL minimal medium plus with 1.75 mM androstenedione, 1.29 mM cholesterol and 5.47 mM glycerol respectively, respectively. CHOL-Up, CHOL-Down, AD-Up and AD-Down correspond to the DEGs up-regulated or down-regulated by CHOL or AD when compared with GL.

To analyze the response to different supplemental carbon sources, *M.* *smegmatis* MC^2^ 155 cultures grown in liquid minimal media supplemented with androstenedione (AD), cholesterol (CHOL) or glycerol (GL) were collected for RNA-Seq. A total of 1.41 giga base pairs (Gb) of paired-end (PE) 90 bp clean reads were obtained from the three sequenced samples (Table 1). The clean reads data are deposited in NCBI’s Sequence Read Archive (SRA) under the accession numbers of SRR1976255, SRR1976286 and SRR1976288. All reads were aligned to the *M.* *smegmatis* MC^2^ 155 genome sequence with Bowtie2 (v2.2.3) [31,32]. Based on the mapping results, the number of mapped reads was calculated and then normalized for genome-wide gene expression profiling. Gene annotations and expression level data for each gene were combined and used for the following analyses. The results showed that 3, 237 and 8 genes were specifically expressed in AD, CHOL and GL (Figure 1, Table S1). The 3 AD-specific genes were all annotated as hypothetical protein encoding genes, whereas the 8 GL-specific genes included seven hypothetical proteins and a rubredoxin encoding gene. GO (Gene Ontology, http://geneontology.org/) classification results showed that 21 genes specifically expressed in CHOL were involved in primary metabolic processes and 15 were involved in nitrogen compound metabolic process. These results indicated that almost all of the genes expressed in androstenedione or glycerol supplemented media were also expressed in cholesterol supplemented media, and that many more genes were induced in cholesterol supplemented media than the other two media.

### 2.2. Differentially Expressed Genes and Functional Enrichment Analyses

To compare the expression patterns between each pairs of conditions, differentially expressed genes (DEGs) with log_2_ fold-change (log_2_FC) ≥1 and False Discovery Rate (FDR) ≤0.05 were identified pair-wisely by edgeR [34]. Results showed that 2019, 489 and 201 DEGs were identified in three comparisons, respectively (Figure 1, Table S2–S4). Compared with AD and GL, a total of 1852 and 454 DEGs were significantly up-regulated in CHOL. Moreover, regardless of the FDR value, a total of 2102 and 1695 genes were up-regulated at least two fold in CHOL when compared with AD and GL, respectively. Among the 1852 significantly up-regulated DEGs, 270 genes were involved in nitrogen compound metabolic processes, 170, 224, 203, 145 and 144 DEGs had oxidoreductase, transferase, hydrolase, nucleic acid binding and ion binding activity, respectively. When compared together, cholesterol appeared to up regulate many more genes than its derivative, androstenedione (Figure 1). A total of 420 genes were up-regulated by cholesterol specifically (CHOL-Up). Most of the CHOL-Up genes were up-regulated by CHOL specifically, including several cholesterol metabolism related genes and some mammalian cell entry related genes (explained further below).

To identify the enriched GO terms, a hypergeometric test was performed and the p-value was adjusted using the Bonferroni method [35]. DEGs identified between AD and CHOL were enriched in the GO term “regulation of response to stimulus”, whereas those identified between CHOL and GL were enriched in “tetrapyrrole binding and oxidoreductase activity”.

To verify the reliability of the RNA-Seq data, we repeated the bacterial cultivation and treatment, re-sampled three biological replicates and confirmed the data by qRT-PCR. qRT-PCR were performed using Ssofast Evagreen supermix (BIO-RAD, Hercules, CA, USA) on a CFX Connect Real-time PCR detection system (BIO-RAD) according to the user manual. A total of 52 genes were selected for qRT-PCR analyses, and 50 fragments were successfully amplified (Table S5, Figure S1). The results demonstrated that most of these genes showed changes consistent with the RNA-Seq data, confirming that the NGS based expression profiling gave reliable expression data in this study.

### 2.3. Nitrogen and Carbon Metabolism Related Genes

**Figure 2:**
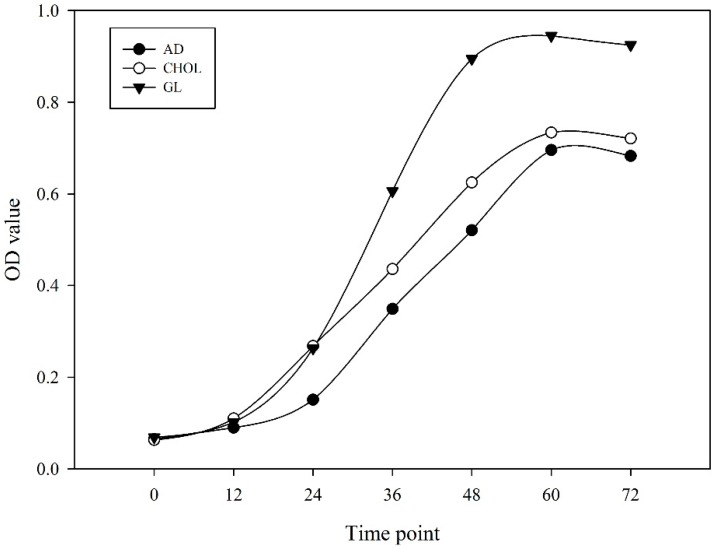
Growth curve of *M.* *smegmatis* MC^2^ 155 grown in different mediums. AD, CHOL and GL correspond to three different conditions: 20 mL minimal medium plus with 1.75 mM androstenedione, 1.29 mM cholesterol and 5.47 mM glycerol respectively.

To detect whether the supplemented carbon source changed basic nitrogen and carbon metabolism, key genes involved in nitrogen and carbon metabolism were carefully analyzed. The assimilation and metabolism of inorganic nitrogen is a complex process involving a series of enzymes, including nitrate reductase (NR, EC:1.7.99.4), nitrite reductase (NiR, EC:1.7.1.4), glutamine synthetase (GS, EC:6.3.1.2), glutamate synthase (GOGAT, EC:1.4.1.13/1.4.1.14/1.4.7.1), glutamate dehydrogenase (GDH, EC:1.4.1.2/1.4.1.3/1.4.1.4), carbamoyl phosphate synthase (CPS, EC:6.3.4.16) *et al.* [36,37]. In this study, *M. smegmatis* strain MC^2^ 155 was grown in chemically defined medium with ammonia-nitrogen as the sole nitrogen source, therefore, the assimilation of ammonia-nitrogen is necessary for maintaining basic metabolism. Three ammonium transporter (*AMT*) encoding genes were analyzed carefully (Table 2). *MSMEG_4635* was the most highly expressed *AMT*, and its expression abundance under different culture conditions was not obviously different between the samples (22.86, 28.85 and 28.80). The absorbed ammonium can be then converted into nitrite by ammonia monooxygenase (EC:1.14.99.39) and hydroxylamine dehydrogenase (EC:1.7.2.6), and then converted into nitrate by NR. However, we could not identify ammonia monooxygenase and hydroxylamine dehydrogenase encoding genes in our RNA-Seq data. Five NR encoding genes were detected and were all highly expressed (Table 2 and Table S1). Among these genes, *MSMEG_2837* was most highly expressed in AD, followed by GL and CHOL, while *MSMEG_5140* and *MSMEG_5139* were most highly expressed in CHOL. The absorbed ammonium can be also catalyzed by CPS, GS and GOGAT to produce carbamoyl phosphate and l-Glutamine. Three CPS encoding genes were all highly expressed in CHOL. Meanwhile, the most highly expressed GS (*MSMEG_4294*) had expression levels of 3744.30, 990.23 and 1818.46 under the three conditions, whereas the other two highly expressed GS genes (*MSMEG_3561* and *MSMEG_4290*) were both most highly expressed in GL. In contrast to the expression pattern of *MSMEG_4294*, the two most highly expressed *GOGATs*, *MSMEG_3225* and *MSMEG_3226*, were both up-regulated by GL, while the other four were all more highly expressed in CHOL. GDH catalyzes the reversible reaction between ammonium and l-Glutamine. However, no obvious difference was observed under different conditions for the most highly expressed GDH encoding gene (*MSMEG_4699*). Furthermore, key enzymes encoding genes involved in glycolysis and the citrate cycle were carefully analyzed, including the hexokinase (HXK, EC:2.7.1.1), 6-phosphofructokinase (PFK, EC:2.7.1.11), pyruvate kinase (PK, EC:2.7.1.40), citrate synthase (CS, EC:2.3.3.1), isocitrate dehydrogenase (IDH, EC:1.1.1.41), alpha-ketoglutarate decarboxylase (KGD, EC:1.2.4.2) and dihydrolipoamide dehydrogenase (DLD, EC:1.8.1.4). Results demonstrated that there were no apparent tendency was observed among three different conditions (Table S1). These results indicated that although different supplements resulted in some changes in gene expression, primary metabolism was not obviously affected. To verify these results, we measured the optical density (OD) value at different time points for three cultivation conditions. It was found that *M.* *smegmatis* MC^2^ 155 showed an S-shaped growth curve in the three different supplemented media (Figure 2).

### 2.4. Glycerol and Androstenedione Metabolism

Glycerol kinase (EC:2.7.1.30) plays a critical role in glycerol metabolism by converting glycerol to glycerol 3-phosphate in an ATP dependent reaction [38]. In this study, we found that three glycerol kinase genes were expressed (Table S1). *MSMEG_6229* was highly expressed under the three different culture conditions, whereas *MSMEG_6756* was expressed at a lower level. *MSMEG_6759* had low expression levels in AD (25.14) and CHOL (8.36), but was significantly up-regulated in GL (456.85). A total of five glycerol-3-phosphate dehydrogenase genes were expressed in this study, and three were highly expressed in all conditions (*MSMEG_1736*, *MSMEG_1140* and *MSMEG_2393*), while *MSMEG_6761* was up-regulated in GL when compared with the other conditions.

Glycerol dehydratase (EC:4.2.1.30) is the rate-limiting enzyme involved in 3-hydroxypropionic acid biosynthesis and glycerol can suppress the activity of glycerol dehydratase [39,40]. Three glycerol dehydratase large subunit encoding genes were identified in our data set (Table S1). *MSMEG_6321* and *MSMEG_0497* had the highest expression level in CHOL, whereas *MSMEG_1547* was highly expressed in all three conditions. However, no glycerol dehydratase medium subunit gene was detected in this study. Four 3-ketosteroid-δ-1-dehydrogenase (EC:1.3.99.4) encoding genes, which are involved in the degradation of androstenedione, were all most highly expressed in CHOL.

### 2.5. Cholesterol Metabolism Related Genes

**Figure 3:**
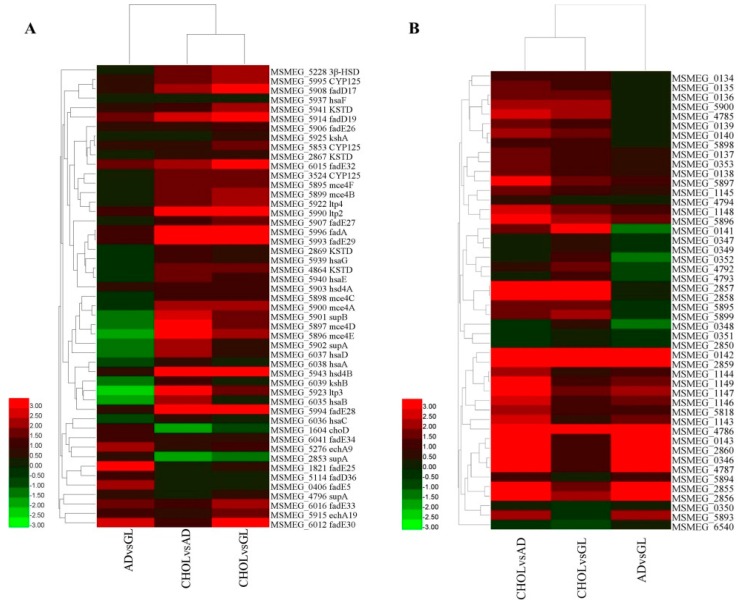
Expression patterns of cholesterol pathway (**A**) and mammalian cell entry (MCE) (**B**) related genes. Log_2_ fold-change (Log_2_FC) was calculated between each two conditions basing on the expression value. Heatmap was drawn by HemI.

In the classic cholesterol catabolism pathway, degradation is initiated by 3-beta hydroxysteroid dehydrogenase/isomerase (3β-HSD) and cholest-4-en-3-one 26-monooxygenase (CYP125, EC:1.14.13.141). In this study, one 3β-HSD encoding gene was found to be expressed (*MSMEG_5228*). When *M. smegmatis* was grown in cholesterol supplemented media, *3β-HSD* was significantly up-regulated, and its expression level was 6.86, 26.34 and 5.96 in AD, CHOL and GL, respectively (Table S6). Three CYP125 encoding genes were detected and all had the highest expression levels in CHOL (*MSMEG_3524, MSMEG_5853* and *MSMEG_5995*) (Figure 3, Table S6). The 3-ketosteroid-δ-1-dehydrogenase (KSTD, EC:1.3.99.4) can catalyze the conversion of androstenedione into androsta-1,4-diene-3,17-dione. When growth media were supplemented with androstenedione or cholesterol *M. smegmatis* strain MC^2^ 155 increased the expression of several *KSTD* genes (*MSMEG_2867*, *MSMEG_2869*, *MSMEG_4864* and *MSMEG_5941*) (Figure 3, Table S6). For example, the expression level of *MSMEG_5941* was 20.86 in GL, but in androstenedione and cholesterol supplemented media, it was 41.15 and 115.42, respectively. *MSMEG_2867* is another KSTD encoding gene that had an expression level of 25.14, 35.54 and 17.88 in AD, CHOL and GL, respectively. 3-Ketosteroid 9-α-hydroxylase (KshAB, EC:1.14.13.142), 3-hydroxy-9,10-secoandrosta-1,3,5(10)-triene-9,17-dione monooxygenase (HsaAB, EC:1.14.14.12), 3,4-dihydroxy-9,10-secoandrosta-1,3,5(10)-triene-9,17-dione 4,5-dioxygenase (HsaC, EC:1.13.11.25) and 4,5:9,10-diseco-3-hydroxy-5,9,17-trioxoandrosta-1(10), 2-diene-4-oate hydrolase (HsaD, EC:3.7.1.17) are the downstream enzymes involved in steroid degradation. However, all of these enzyme encoding genes were not detected based on the annotation information in the genome.

A recent study showed that a gene cluster is involved in the cholesterol catabolism [10], including *KshAB*, *HsaA*, *HsaB*, *HsaC* and *HsaD* genes mentioned above. In this study, these genes were firstly extracted from the genome of *M. tuberculosis* H37Rv and then used as a search query in a sequence similarity BLAST search against the deduced proteome of *M. smegmatis* MC^2^ 155. A total of 26 homologous genes were successfully identified corresponding to 24 H37Rv genes (Figure 3, Table S6), including *kasAB* and *hasA/B/C/D*, which were not previously identified based on the annotation information present in the genome. Expression profiling showed that 24 of these 26 homologous genes were most highly expressed in CHOL, except *choD* and one of the three *supA* genes. Moreover, a *MCE4* gene cluster was identified as being involved in cholesterol catabolism and was significantly up-regulated in the cholesterol supplemented sample. Compared with AD, these *MCE4* cluster genes were up-regulated at least 2.72-fold, whereas that was 2.19-fold when compared with GL. Griffin *et al.* used high-resolution phenotypic profiling to reveal genes essential for growth with cholesterol in *M. tuberculosis* [41], including the genes involved in the catabolism of the side-chain and several other essential genes. Besides CYP125, a total of 15 side-chain degradation related genes were also identified in this study based on BLAST similarity search (Table S6), and most of them were most highly expressed in cholesterol supplemented media, except *fadD36*, *fadE5*, *fadE25*, *fadE34* and *echA9*. In contrast to the report of Griffin *et al.*, *hsd4B* of *M. smegmatis* MC^2^ 155 was successfully detected and significantly up-regulated by cholesterol (18.29, 87.82 and 27.81 in AD, CHOL and GL). Sixty other genes, corresponding to 53 essential genes for growth on cholesterol listed by Griffin *et al.*, were carefully analyzed. However, most of these did not show obvious differences among the three growth conditions (or were not up-regulated by cholesterol).

### 2.6. Mammalian Cell Entry Related Genes

**Figure 4:**
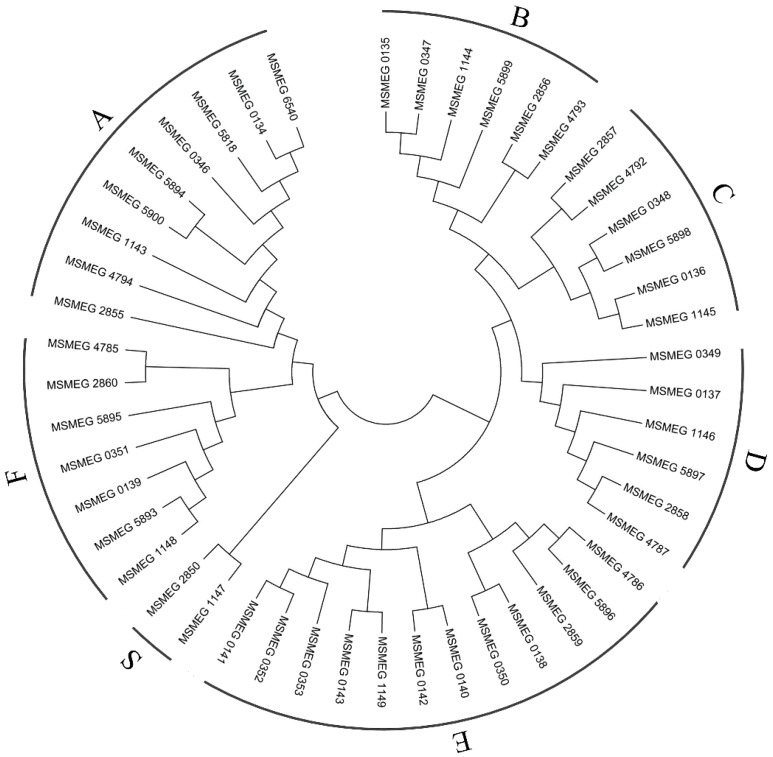
Phylogenetic analysis of 48 mammalian cell entry (MCE) related proteins. Protein sequences were deduced from 48 MCE related genes. Phylogenetic tree was constructed using the Neighbor–Joining method in MEGA6 program [43]. Group A, B, C, D, E, F are consisted of *MCE A*, *B*, *C*, *D*, *E*, *F* genes, respectively, group S means an additional group.

Mammalian cell entry (MCE) is a protein family that is crucial for virulence of certain members of the genus *Mycobacterium*, which enables mycobacteria to derive carbon and energy from the cholesterol of host cell membranes and to enter mammalian cells [42]. In this study, 48 MCE related genes were detected. Combining the genome annotation results with the BLASTP similarity search results, a total of 11 *MCE1*, one *MCE2*, six *MCE3*, six *MCE4* and six *MCE5* genes were identified. To further analyze these MCE related genes, a phylogenetic tree was constructed using the Neighbor-Joining method in the MEGA6 program [43] (Figure 4). It was shown that the 48 MCE related genes can be clustered into seven groups, including group A–F, as well as an additional group (Figure 4, Table S7). The A group consisted of nine *MCE A* genes, B, C and D group had six *MCE B*, *MCE C* and *MCE D* separately, E and F group consisted of 12 and seven genes. The additional group (S) consisted of *MSMEG_2850* and *MSMEG_1147*, which were annotated as *MCE5E* and MCE related family protein, respectively (Figure 4, Table S7).

**Figure 5:**
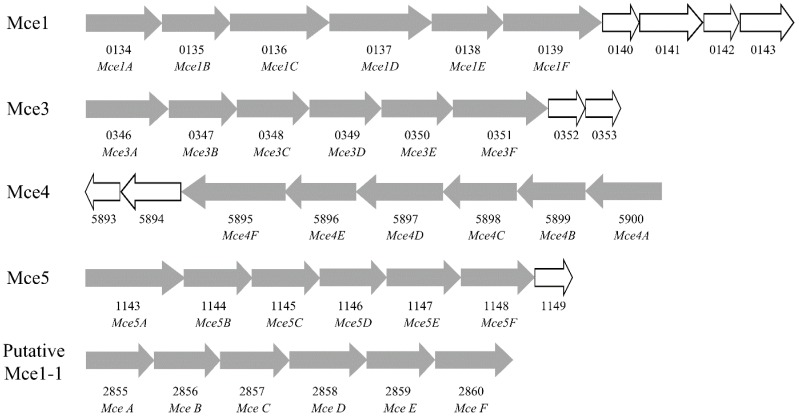
The organization of the *MCE* operons in *M. smegmatis* MC^2^ 155 genome. The operon structures of the five *MCE* operons are shown. The grey arrows represent *MCE* genes, while the white arrows represent other *MCE* related genes.

Expression analyses showed that 43 of the 48 MCE related genes were most highly expressed in CHOL, whereas 11 of the 48 MCE genes were not expressed in AD, including the genes encoded by putative *MCE1* operon. Forty-six MCE related genes were expressed in GL, and most of these had higher expression levels than in AD (Figure 4, Table S7). *MSMEG_2857* and *MSMEG_2858* were specifically expressed in CHOL, with an expression level of 12.55 and 12.96, respectively. According to the *M.* *smegmatis* MC^2^ 155 genome annotation, *MSMEG_2857* and *MSMEG_2858* are both annotated as virulence factor MCE family proteins, but the phylogenetic analysis showed that they were separately clustered into groups C and D. The location of the 48 genes demonstrated that there are at least five *MCE* operons, including *MCE1*, *MCE3*, *MCE4*, *MCE5* and a putative *MCE1* operon (Figure 5). Apart from *MCE4*, all other operons were encoded on the plus strand. The expression levels of *MSMEG_0134* to *MSMEG_0139*, which encode *MCE1A* to *MCE1F*, were all higher than 800 in CHOL (Table S7), indicating that the corresponding *MCE1* operon should be dominant. According to the present RNA-Seq data, the expression values of the MCE4ABCDEF encoding genes (*MSMEG_5900*, *MSMEG_5899*, *MSMEG_5898*, *MSMEG_5897*, *MSMEG_5896*, and *MSMEG_5895*) were at a basal level in GL and AD (Table S7), and were up-regulated by cholesterol. The different expression levels of these operons reflected their different roles.

### 2.7. Gene Clusters Induced by Cholesterol

Uhía *et al.* [27] described three gene clusters in *M.* *smegmatis* MC^2^ 155 that were induced by cholesterol when compared with glycerol. In this study, these gene clusters, including cluster 1 (*MSMEG_5990*–*6017*), cluster 2 (*MSMEG_6033*–*6043*) and cluster 3 (*MSMEG_5903*–*5943*), were also analyzed and most of these genes were up-regulated by cholesterol compared with that in glycerol supplemented medium. Moreover, most of these genes were also up-regulated by cholesterol when compared with androstenedione, the derivative of cholesterol (Table S1). As they have greater throughput, RNA-Seq methods can quantify more genes than microarray technology [30]. With threshold of log_2_FC ≥ 1, it was found that another eight gene clusters were induced by cholesterol when compared with glycerol, and another 13 gene clusters were induced by cholesterol compared with androstenedione (Table 3). The gene clusters *MSMEG_0132*–*0144*, *MSMEG_1141–1150*, *MSMEG_2854–2865* and *MSMEG_4414–4427* were induced by cholesterol when compared with either androstenedione or glycerol. Results showed that the four clusters all encoded MCE related genes. *MSMEG_0500*–*0518* was found to encode several proteins related to carbohydrate metabolism, *MSMEG_0638*–*0649* encodes several transporters, and *MSMEG_1435*–1448 encodes several ribosomal proteins.

## 3. Discussion

### 3.1. Genome-Wide Transcriptome Changes Response to Different Supplements

Uhía *et al.* found that 89 *M. smegmatis* genes were up-regulated at least three fold during growth on cholesterol compared with growth on glycerol by using microarray [27]. Microarray usually show limitations on throughput, while RNA-Seq has been proved to be a cost effective technology to characterize genome-wide transcription and has been widely used since its inception in 2005 [29,44,45]. Recently, RNA-Seq technology has also been used to detect the nitrogen limitation response and the GlnR regulon in *M.* *smegmatis* [46]. However, no systematic analysis has been performed to investigate the genome-wide cholesterol response in this model bacterium by using RNA-Seq technology. In this study, Illumina sequencing was employed to comprehensively reveal the cholesterol response in *M.* *smegmatis* MC^2^ 155. Expression patterns of genes related to nitrogen and carbon metabolism were carefully analyzed, and although occasional changes were detected, no apparent tendency can be observed among three samples (Table S1). Moreover, *M.* *smegmatis* MC^2^ 155 showed an S-shaped growth curve in androstenedione, cholesterol and glycerol supplemented media (Figure 2). These results suggested that *M.* *smegmatis* MC^2^ 155 grew well in minimal medium with different supplements. Differential expression analyses showed that a total of 1852 and 454 genes were significantly up-regulated by cholesterol when compared with androstenedione and glycerol, respectively, and 237 genes were specifically expressed in cholesterol supplemented medium. The majority of genes in the three cholesterol induced clusters described by Uhía *et al.* [27] were also up-regulated by cholesterol, when compared with that in glycerol or androstenedione supplemented media. Besides, a total of 13 and eight new clusters were found to be induced by cholesterol when compared with the other two supplements, respectively (Table 3). These observations above showed that cholesterol up-regulated and induced a large number of genes, but androstenedione did not stimulate the same response. The gene clusters identified in this study should provide a new resource for further studies focusing on cholesterol metabolism.

The minimal medium used in this study contained all macroelements, including carbon, nitrogen, phosphorus, sulphur and magnesium, among others, which are sufficient to maintain basic metabolism and growth. With different molecular structure and a different number of carbon atoms, supplementation with androstenedione, glycerol and cholesterol modified the expression patterns of several genes or some genes involved in special metabolic pathways, such as glycerol, androstenedione and cholesterol metabolism (Figure 4, Table S1).

### 3.2. Cholesterol Catabolism and Mammalian Cell Entry Related Genes

Tak [47] and Turfitt [48] confirmed that mycobacteria are able to decompose cholesterol and that some mycobacteria can grow in medium with cholesterol as sole carbon source. Mycobacteria use different parts of the cholesterol molecule for energy and the biosynthesis of phthiocerol dimycocerosate (PDIM) [6]. *MCE* is a key gene family involved in this metabolic process and has been shown to be critical for the survival of *M. tuberculosis* in the macrophage [9,10]. It is a highly conserved gene family, which is widely distributed in the genus of *Mycobacterium*, including the non-pathogenic mycobacteria *M.* *smegmatis* [41,49]. Different from *M. tuberculosis* [6], 48 *MCE* genes were found to be distributed in at least five *MCE* operons in the genome of *M.* *smegmatis* MC^2^ 155 (Figure 5). For each operon, at least six core genes (*MCE A-F*) were found to be expressed. However, *MSMEG_4785*, *MSMEG_4786*, *MSMEG_4787*, *MSMEG_4792*, *MSMEG_4793* and *MSMEG_4794* may be encoded by another operon, although *MSMEG_4788*, *MSMEG_4789*, *MSMEG_4790* and *MSMEG_4791* are annotated as non-MCE protein encoding genes. The 48 *MCE* genes were all expressed in cholesterol supplemented medium, and almost all of these *MCE* genes were up-regulated by cholesterol when compared with the other supplements (Figure 4). Particularly, the putative *MCE1* operon encoded six genes were not expressed in androstenedione supplemented medium. Among the different *MCE* operons, the *MCE4* operon has been characterized as an efficient cholesterol uptake system [6,12]. In *M. tuberculosis*, proteins encoded by these genes are implicated in the interaction of this pathogen with its human host. Therefore, the increased expression of the *MCE4* operon in this study may allow more cholesterol to be transported into the cell and promote the expression of other MCE related genes. In *Gordonia neofelifaecis*, genes in the *MCE4* operon showed low differential expression in cholesterol supplemented medium [50]. But in this study, the *MCE4* genes were significantly up-regulated in cholesterol (more than 2-fold), and this was confirmed by quantitative real-time PCR (qRT-PCR).

MCE1 is considered to be involved in the import of fatty acids, but its role is still controversial, as the precise substrate transported by the MCE1 proteins and the contribution of this transporter to intracellular growth are still poorly understood. Several groups have investigated the expression of the *MCE1* operon, and the results obtained by different groups are conflicting. Quantitative reverse transcriptase PCR analyses conduct by Casali *et al.* revealed that the *MCE1* genes in *M. tuberculosis* are expressed during *in vitro* growth, but are significantly down-regulated in intracellular bacilli isolated from murine macrophages [51], and Kumar reported that the *MCE1* operon is up-regulated in bacilli isolated from both mouse and rabbit lungs [52]. In this study, *MCE1A*, *B*, *C*, *D*, *E*, *F* and a putative *MCE1* operon encoding genes were found to be up-regulated by cholesterol compared with the other two culture conditions. As a derivative of cholesterol without alkyl side-chain, androstenedione shares its ring A and B structure with cholesterol. However, androstenedione did not improve the expression of *MCE1* genes. This can be partially explained by the lack of an efficient uptake system for androstenedione, as the *MCE4* system can only recognize a side-chain with at least eight carbons [12]. Consequently, these results indicated that ring C and D or the aliphatic chain of cholesterol (that did not exist in androstenedione molecular structure) may play a crucial role in promoting the expression of these *MCE* genes. The alkyl side chain is degraded by a process similar to the β-oxidation of fatty acids and proceeds via CoA thioester intermediates. The absorbed cholesterol would then serve as a source of propionate *in vivo* and results in a sufficient intracellular pool of propionate to increase methyl-branched fatty acid biosynthesis [53].

Previous studies have found that 41 of the cholesterol degradation pathway genes of *M. tuberculosis*, including genes involved in the uptake system, catabolism initiation and catabolism of rings A/B/C/D, are among those specifically up-regulated during survival in the macrophage [10,54]. The majority of these genes were also identified in our *M.* *smegmatis* MC^2^ 155 RNA-Seq data and were found to be up-regulated (except *choD* and one of the three *supA* gene) in cholesterol supplemented media when compared with the other supplements (Figure 4, Table S6). ChoD is reported to be involved in the initiation of cholesterol catabolism, and may act extracellularly or be associated with the cell-surface in some Mycobacterium strains [14,15]. The low expression level of *choD* in cholesterol supplemented medium may be due to *choD* being non-essential for growth in cholesterol for *M.* *smegmatis* MC^2^ 155, as has been shown for *M. tuberculosis* H37Rv [41]. In fact, it has been demonstrated that *choD* found in some Mycobacteria does not play role in cholesterol degradation [41,55]. When grown in androstenedione supplemented medium, *choD* was highly expressed, indicating that *choD* may be involved in the initiation of the extracellular catabolism of androstenedione. However, this hypothesis requires further investigation. The other gene involved in the initiation of cholesterol catabolism, 3β-HSDs [56], was up-regulated by cholesterol, suggesting that the catabolism initiation can be catalyzed by 3β-HSDs in *M.* *smegmatis* MC^2^ 155. However, genes involved in the degradation of rings A and B, including those that encode KstD, KshAB, HsaA, HsaB, HsaC and HsaD [2,57,58,59,60,61], were not up-regulated by androstenedione when compared with that in glycerol supplemented medium. This can also be partially explained by the lack of an efficient uptake system for androstenedione. In the study of Brzostek *et al.* in 2005 [22], a total of six putative KstD genes were described. However, all of these gene ID (*MSMEG_2871*, *2873*, *4850*, *4855*, *5801*, *5898*) are no longer annotated as KstD in the improved genome annotation, but are annotated as a hypothetical protein, hypothetical protein, short-chain dehydrogenase/reductase (SDR), amidohydrolase, hydroxylase and virulence factor MCE family protein, respectively, which should be the results of improvement of genome annotation. According to the DEGs analyses, steroid-degrading genes showed a low differential expression in androstenedione supplemented medium compared with glycerol. Literatures showed that a TetR-type transcriptional repressor named KstR controls the expression of 83 catabolic genes, which might be involved in steroid degradation [62]. It was reported that the inducer of KstR in *M. smegmatis* MC^2^ 155 is 3-oxo-4-cholestenoic acid, the first metabolic intermediate in cholesterol degradation, which binds KstR and results in up-regulation of steroid-degrading genes [63]. Compared with cholesterol, degradation of androstenedione and glycerol could not produce 3-oxo-4-cholestenoic acid, and this might be the reason that steroid-degrading genes were not up-regulated during growth on androstenedione compared with glycerol (Table S6). In consideration of different molecular structure of cholesterol and androstenedione, these results indicated that the side-chain mediated uptake play crucial role in stimulating the expression of cholesterol degradation related and some other genes. Griffin *et al.* used high-resolution phenotypic profiling to reveal genes essential for growth in cholesterol in *M. tuberculosis* [41]. Eighteen side-chain degradation related genes were also identified based on BLAST similarity searches (Table S6). However, some of those genes were not up-regulated by cholesterol in this study. As several degradation reactions can be catalyzed by different enzymes [41], functional redundancy should be the most likely reason. For example, *fadD19*, *36* are involved in the same reaction, but only *fadD19* was up-regulated.

In this study, we did not use biological replicates but tried our best to minimize the sampling deviation by collecting *M. smegmatis* MC2 155 strains from three culture replicates. To verify the reliability of the RNA-Seq data, we repeated the bacterial cultivation and treatment, re-sampled three biological replicates and confirmed the data with qRT-PCR. Twenty-six of the 32 genes described above had the highest expression level in CHOL (Figure S1). Although there is still a small number of genes whose expression was inconsistent, such as *MSMEG_2869*, *MSMEG_3561*, *MSMEG_4290*, the consistently expressed genes between of the two quantitative technologies proved the trends observed in this study. Therefore, we can conclude that cholesterol increased the expression of most cholesterol degradation and *MCE* involved genes in *M. smegmatis*.

## 4. Materials and Methods

### 4.1. Bacterial Strains

*M.* *smegmatis* MC^2^ 155 is a mutant of *M. smegmatis* [26]. It is invaluable in analyses of mycobacterial gene function, expression and replication due to its efficiently plasmid transformation rate. The strains used in this study were preserved in our laboratory.

### 4.2. Bacterial Cultivation and Sampling

*M.* *smegmatis* MC^2^ 155 was pre-cultured in 2 mL Luria-Bertan (LB) to OD_600_ = 1.0, then the cells were centrifuged down, collected and washed twice with minimal medium (1.5 g CH_3_COONH_4_, 0.2 g MgSO_4_·7H_2_O, 0.4 g K_2_HPO_4_, 0.8 g KH_2_PO_4_, 5.0 × 10^−4^ FeSO_4_·7H_2_O, 2.0 × 10^−4^ ZnSO_4_·7H_2_O, 5.0 × 10^−5^ MnCl_2_·4H_2_O). The cultures were subsequently divided into three groups: one was grown in 20 mL minimal medium plus with 1.75 mM (0.05%) androstenedione (AD) (Sigma, St. Louis, MO, USA), one in 20 mL minimal medium plus with 1.29 mM (0.05%) cholesterol (CHOL) (Sigma, St. Louis, MO, USA), and one in 20 mL minimal medium plus with 5.47 mM (0.05%) glycerol (GL) (Sigma, St. Louis, MO, USA). For optimum mixing of cholesterol and androstenedione in water, a certain amount of the steroid substrates were firstly mixed with cyclodextrin, and added to 5 mL of solution containing 50% Tween-80, sonicated for 30 min, then added to the minimal medium, and sonicated for a further 30 min. The final medium had a concentration of 0.9 mM cyclodextrin and 0.25% (*v*/*v*) Tween-80. The control sample containing glycerol also contained 0.9 mM of cyclodextrin and 0.25% of Tween-80. All of these *M.* *smegmatis* MC^2^ 155 were cultivated at 30 °C in an orbital shaker at 180 rpm for 48 h. Three cultures were marked as AD, CHOL and GL.

### 4.3. RNA Extraction and Library Construction

Total RNA was isolated from *M.* *smegmatis* MC^2^ 155 samples with the RNeasy Mini Kit (50) (Qiagen, Hilden, Germany) and then treated with the DNase I RNase-Free DNase Set (Qiagen) to remove genomic DNA contamination according to the manufacture’s protocols. For each sample, *M.* *smegmatis* MC^2^ 155 strains were collected from three culture replicates and pooled together following RNA extraction, in order to minimize the sampling deviation. rRNA was removed by Ribo-Zero_rRNA Removal Kit (Epicentre Biotechnologies, Madison, WI, USA). The purity, concentration and RNA integrity number (RIN) were evaluated by Nanodrop ND-2000 (Nanodrop, Wilmington, NC, USA). The qualified mRNA was then fragmented in fragmentation buffer. Taking these short fragments as templates, random hexamer-primers were used to synthesize the first-strand cDNA. The second strands were synthesized using buffer, dNTPs, RNase H and DNA polymerase I. Short fragments were purified with the QiaQuick PCR extraction kit (Qiagen) and resolved with EB buffer for end repair. The short fragments were then ligated to Illumina sequencing adaptors. Fragments with different lengths were separated by agarose gel electrophoresis. The 200 bp cDNAs fragments were purified from a gel and used for the following template enrichment by PCR with two primers that anneal to the end of the sequencing adapters. Quality control analysis of the fragmented cDNA library was performed on an Agilent 2100 Bioanalyzer (Agilent Technologies, Palo Alto, CA, USA).

### 4.4. RNA Sequencing and Data Analyzing

The validated 200 bp fragment cDNA libraries were submitted to the Illumina Hiseq 2000 platform at Beijing Genome Institute (BGI, Shenzhen, China) to perform transcriptome sequencing. The Illumina sequencing by-synthesis, image analysis and base-calling procedures were used to obtain paired-end (PE) reads and base-calling quality values. The raw reads were cleaned by removing the reads with adapter sequence or excessive “N” bases (more than 10%), as well as low quality reads, for which the percentage of low quality bases is over 20% in a read. PE reads were then mapped to the entire genome of *M.* *smegmatis* MC^2^ 155 [64] with Bowtie2 (v2.2.3) allowing no more than 1 mismatche in a read [31,32]. Based on the mapping results, reads mapped to each gene was calculated and used for the following expression profiling. The annotation information of the genome project and the expression value of each gene were combined and then used. The significant differentially expressed genes (DEGs) with log2 fold-change (log2FC) ≥ 1 and FDR ≤ 0.05 were identified between each pair of conditions by edgeR [34]. For each DEGs set, a hypergeometric test was performed and the p-value was adjusted using the Bonferroni method [35]. Compared with the reference genome, significantly enriched GO (Gene Ontology, http://geneontology.org/) terms and KEGG (Kyoto Encyclopedia of Genes and Genomes) [65] pathway were screened with threshold of corrected *p*-value (padj) ≤0.05.

## Figures and Tables

**Table 1:** RNA-Sequencing read mapping. Androstenedione (AD), cholesterol (CHOL) and glycerol (GL) correspond to three different conditions: 20 mL minimal medium plus with 1.75 mM (0.05%) androstenedione, 1.29 mM (0.05%) cholesterol and 5.47 mM (0.05%) glycerol respectively. RNA-Seq reads were mapped to the genome and rRNA of *M.* *smegmatis* MC^2^ 155 with Bowtie2 (v2.2.3) [31,32].

Read Classification	AD	CHOL	GL
Total Reads	4,727,962	5,524,220	5,432,638
Mapped	4,630,566	5,403,240	5,325,615
Unmapped	97,396	120,980	107,023
rRNA Mapped	38,769	28,174	19,557

**Table 2:** Expression level of nitrogen and carbon metabolism, and also glycerol and androstenedione metabolism related genes.

Gene Name	Gene_ID	Expression Level		
		Myco_1	Myco_2	Myco_3
ammonium transporter	*MSMEG_4635*	22.86	28.85	28.80
nitrate reductase	*MSMEG_2837*	806.92	166.85	351.58
nitrate reductase	*MSMEG_5139*	107.44	326.17	183.73
nitrate reductase	*MSMEG_5140*	210.30	614.29	442.95
carbamoyl phosphate synthase	*MSMEG_3046*	6.86	37.22	12.91
carbamoyl phosphate synthase	*MSMEG_3047*	34.29	73.60	35.75
carbamoyl phosphate synthase	*MSMEG_4726*	0.00	0.42	0.00
glutamine synthetase	*MSMEG_3561*	393.17	392.24	640.58
glutamine synthetase	*MSMEG_4290*	416.03	477.55	685.27
glutamine synthetase	*MSMEG_4294*	3744.30	990.23	1818.46
glutamate dehydrogenase	*MSMEG_4699*	1769.28	1946.59	1900.89
glutamate synthase	*MSMEG_6263*	29.72	36.80	23.84
glutamate synthase	*MSMEG_6458*	4.57	23.00	5.96
glutamate synthase	*MSMEG_3226*	240.02	117.92	295.96
glutamate synthase	*MSMEG_3225*	985.22	484.24	1505.62
glutamate synthase	*MSMEG_5594*	22.86	82.38	26.82
glutamate synthase	*MSMEG_6459*	25.14	70.67	30.79
hexokinase	*MSMEG_5577*	313.17	225.81	397.26
6-phosphofructokinase	*MSMEG_2366*	1430.97	288.54	746.85
pyruvate kinase	*MSMEG_3227*	1312.10	1222.31	2115.41
citrate synthase	*MSMEG_5676*	598.90	155.14	395.27
isocitrate dehydrogenase	*MSMEG_1654*	1675.56	1218.55	1752.91
alpha-ketoglutarate decarboxylase	*MSMEG_5049*	1398.97	2698.88	3475.03
dihydrolipoamide dehydrogenase	*MSMEG_0903*	612.62	425.70	414.14
glycerol kinase	*MSMEG_6759*	25.14	8.36	456.85
glycerol kinase	*MSMEG_6229*	294.88	271.81	195.65
glycerol kinase	*MSMEG_6756*	0.00	12.55	12.91
glycerol-3-phosphate dehydrogenase	*MSMEG_1736*	651.48	705.87	637.60
glycerol-4-phosphate dehydrogenase	*MSMEG_6761*	64.01	38.05	302.91
glycerol-5-phosphate dehydrogenase	*MSMEG_1140*	777.20	1007.79	1057.71
glycerol-6-phosphate dehydrogenase	*MSMEG_2393*	182.87	110.82	244.32
glycerol dehydratase	*MSMEG_0497*	0.00	30.11	8.94
glycerol dehydratase	*MSMEG_1547*	246.88	221.63	262.19
glycerol dehydratase	*MSMEG_6321*	48.00	120.85	57.60
3-ketosteroid-δ-1-dehydrogenase	*MSMEG_2867*	25.14	35.54	17.88
3-ketosteroid-δ-2-dehydrogenase	*MSMEG_2869*	9.14	18.40	10.92
3-ketosteroid-δ-3-dehydrogenase	*MSMEG_4864*	4.57	17.15	5.96
3-ketosteroid-δ-4-dehydrogenase	*MSMEG_5941*	41.15	115.42	20.86

**Table 3:** Gene clusters induced by cholesterol. AD, CHOL and GL correspond to three different conditions: 20 mL minimal medium plus with 1.75 mM androstenedione, 1.29 mM cholesterol and 5.47 mM glycerol respectively.

CHOLvsAD		CHOLvsGL	
Start	End	Start	End
*MSMEG_0132*	*MSMEG_0144*	*MSMEG_0132*	*MSMEG_0144*
*MSMEG_0329*	*MSMEG_0346*	*MSMEG_2705*	*MSMEG_2714*
*MSMEG_0500*	*MSMEG_0518*	**	**
*MSMEG_0638*	*MSMEG_0649*	**	**
*MSMEG_1141*	*MSMEG_1150*	*MSMEG_1141*	*MSMEG_1150*
*MSMEG_1364*	*MSMEG_1375*		
*MSMEG_1435*	*MSMEG_1448*		
*MSMEG_2705*	*MSMEG_2713*	*MSMEG_6115*	*MSMEG_6125*
*MSMEG_2854*	*MSMEG_2865*	*MSMEG_2854*	*MSMEG_2861*
*MSMEG_3997*	*MSMEG_4005*		
*MSMEG_4414*	*MSMEG_4427*	*MSMEG_4419*	*MSMEG_4427*
*MSMEG_4835*	*MSMEG_4843*	*MSMEG_4461*	*MSMEG_4468*
*MSMEG_5953*	*MSMEG_5966*	*MSMEG_4864*	*MSMEG_4873*

## Supplementary Materials

Supplementary materials can be found at http://www.mdpi.com/1422-0067/17/5/689/s1.
