# Supplementary material for: Genome-Wide Transcriptome Profiling of Mycobacterium smegmatis MC2 155 Cultivated in Minimal Media Supplemented with Cholesterol, Androstenedione or Glycerol
Source: Int J Mol Sci. 2016 May 7;17(5):689. doi: 10.3390/ijms17050689 (PMC4881515; doi:10.3390/ijms17050689)
Supplement: Supplementary file 1 [file ijms-17-00689-s001.zip › ijms-121984-Supplementary Materials/ijms-121984-Figure S1.pdf]

Supplementary Materials: Genome-Wide Transcriptome Profiling of *Mycobacterium smegmatis* MC² 155 Cultivated in Minimal Media Supplemented with Cholesterol, Androstenedione or Glycerol

Qun Li, Fanglan Ge, Yunya Tan, Guangxiang Zhang and Wei Li

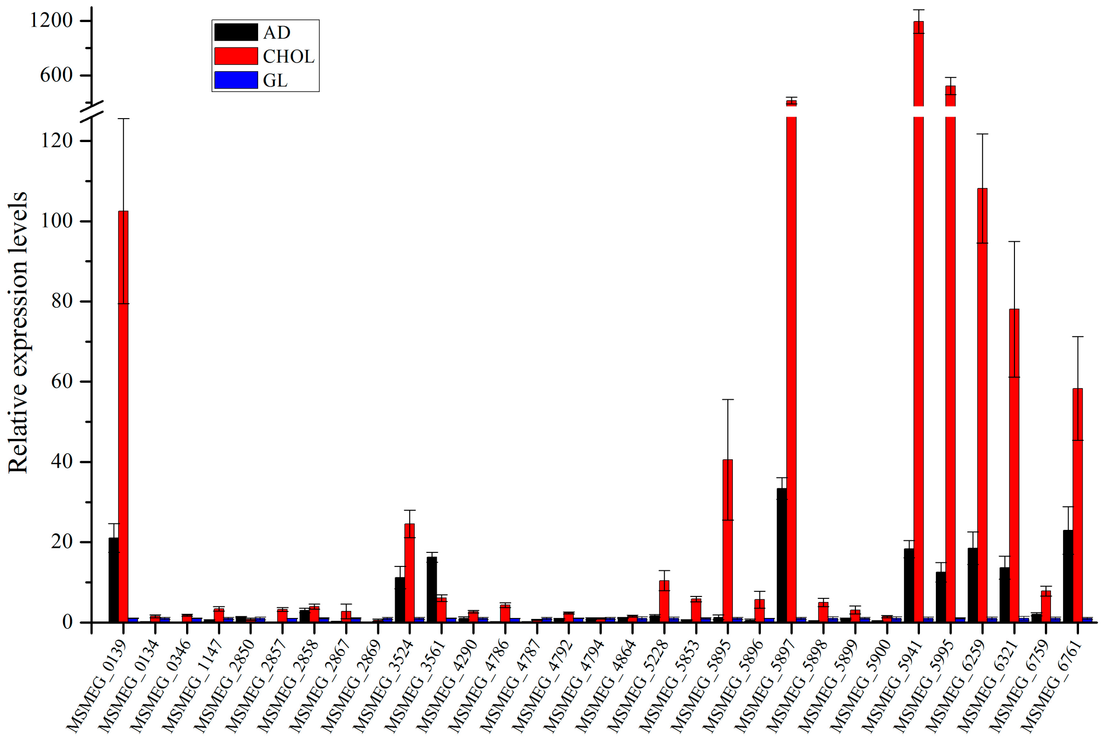

Figure S1. qRT-PCR results of some selected genes.
